# Duodenal varices and evaluation of endoscopic cyanoacrylate injection for treatment of duodenal variceal bleeding

**DOI:** 10.1016/j.iliver.2025.100143

**Published:** 2025-01-17

**Authors:** Jin-dong Chu, Qian Bi, Yan-ling Wang, Xue-mei Ma, Bo Liu, Liang Wu, Shuai Wang, Li-jun Shen, Xiao-bao Qi, Zheng Lu

**Affiliations:** Senior Department of Hepatology, the Fifth Medical Center of PLA General Hospital, Beijing 100039, China

**Keywords:** Portal hypertension, Duodenal varices, Cyanoacrylate, Endoscopic treatment

## Abstract

**Background and aims:**

Duodenal varices (DVs) are a rare complication of portal hypertension. This study analyzed the clinical characteristics of DVs and examined the efficacy of endoscopic cyanoacrylate injection for duodenal variceal bleeding.

**Methods:**

The clinical data of patients with DVs treated in our hospital from March 2013 to May 2024 were retrospectively analyzed.

**Results:**

During the study period, 80,850 patients underwent gastroscopy for a total of 122,040 endoscopy sessions. DVs were diagnosed in 52 patients. Eight patients with DVs exhibited duodenal variceal bleeding (15.4%). The overall prevalence of DVs was 0.08%.The prevalence of DVs among patients with upper gastrointestinal varices was 0.16%. The most common etiology of DVs was liver cirrhosis (92.3%). DVs location was the descending segment of the duodenum in 34 patients (65.4%). Forty-four patients (84.6%) had concomitant esophageal and gastric varices. Successful hemostasis was achieved at the time of endoscopy in all patients undergoing emergency endoscopic treatment using cyanoacrylate injection. The 6-week mortality rate was 12.5%. The rebleeding rate was 12.5%.

**Conclusions:**

DVs are uncommon, even in hospitals where liver disease is prevalent. Emergency endoscopic cyanoacrylate embolization appears to be highly effective. Complete vein embolization may be considered for patients in poor condition. Age >65 years and low hemoglobin concentration are predictors of duodenal variceal bleeding.

## 1 Introduction

Portal hypertension in patients with liver cirrhosis frequently leads to the development of portosystemic collateral circulation, which can result in gastrointestinal varices. Duodenal varices (DVs) are an uncommon manifestation of portosystemic collateral circulation. In one study, the endoscopic detection rate of DVs was 0.2% in patients who underwent gastroscopy screening and the incidence of DVs in patients with portal hypertension was 0.4%.^1^ In a large single-center study in China, the overall prevalence of DVs was 0.34 per 10,000, and DVs accounted for only 0.13% of all gastrointestinal varices.^2^

Esophagogastric variceal bleeding is a major etiology of upper gastrointestinal bleeding in patients with portal hypertension. Endoscopic approaches are currently the first line of treatment for acute esophagogastric variceal bleeding; they are also useful for secondary prophylaxis of bleeding. Baveno Ⅶ recommended band ligation for esophageal variceal bleeding, band ligation and tissue adhesive (e.g., N-butyl-cyanoacrylate) for type 1 gastroesophageal varices, and tissue adhesive for type 2 gastroesophageal varices and isolated gastric varices.^3^

Owing to the low incidence of DVs, there are few studies regarding the treatment of duodenal variceal bleeding. Endoscopic treatment, transjugular intrahepatic portal systemic shunt, and balloon-occluded retrograde transvenous obliteration are the main treatments.^4^, ^5^, ^6^, ^7^ Endoscopic treatment is more widely used because of its minimally invasive nature. Baveno VII recommended that either endovascular or endoscopic treatment be considered in patients with ectopic varices.^2^ Cyanoacrylate is a monomer-based tissue adhesive that instantly polymerizes and rapidly solidifies upon contact with blood, which makes it an effective embolic agent. A recent systematic review suggested that endoscopic tissue adhesive may be a preferable treatment for duodenal variceal bleeding.^7^

This study retrospectively analyzed patients with DVs detected via endoscopy at our hospital and investigated the efficacy and safety of endoscopic cyanoacrylate injection for treating duodenal variceal bleeding.

## 2 Patients and methods

### 2.1 Patients

Patients who were diagnosed with DVs via endoscopy and endoscopic ultrasound at our hospital from March 1, 2013 to May 31, 2024 were eligible for inclusion. Demographic and clinical data were collected from the medical record. Endoscopic duodenal variceal bleeding was defined as active bleeding, thrombus, or erosion of DVs.

GIF-Q260 and GIF-Q290 endoscopes (Olympus Optical Corporation, Tokyo, Japan) were used for endoscopy. Endoscopic control of duodenal variceal bleeding was obtained with injection of 50% glucose and N-butyl-cyanoacrylate (Beijing Compont Medical Devices Co. Ltd., Beijing, China) through a 23 or 25-gauge needle (MTW Company, Germany) using the classic ‘sandwich’ method. The dose of cyanoacrylate used was based on hemostasis efficacy, and the aim of treatment was hemostasis, not complete embolization of all varices. Secondary prophylaxis was administered to patients whose DVs were not completely embolized and who were classified as Child‒Pugh class A; in these patients, the procedure was the same but the aim of treatment was complete embolization. Ceftriaxone was routinely administered. Patients were clinically followed for 6 weeks after treatment. Those who experienced rebleeding after emergency endoscopic treatment underwent repeat endoscopic treatment. All treatments were performed by experienced endoscopists. Outpatient or telephone follow-up was conducted to obtain data on treatment effects and adverse events.

### 2.2 Ethics committee approval

Written informed consent was obtained from all patients. The study protocol was approved by the institutional review board of the Fifth Medical Center of Chinese PLA General Hospital (KY-2024-7-98-1).

### 2.3 Definitions

Successful hemostasis was defined as the cessation of active bleeding during therapeutic endoscopy, stable vital signs, no decrease in hemoglobin concentration, and no recurrence of bleeding within 24 h.^8^ Treatment failure was defined as either the absence of hemostasis or rebleeding within the first 5 days.^3^ Rebleeding was defined as either a single or repeated occurrence of hematemesis or melena after 5 days which resulted in any of the following: (1) hospitalization, (2) blood transfusion, (3) decrease in hemoglobin of 3 g, or (4) death.^8^ Acute-on-chronic liver failure was defined as an acute hepatic insult that manifests with jaundice (serum bilirubin ≥5 mg/dL or 85 μmol/L) and coagulopathy (international normalized ratio ≥1.5 or prothrombin activity <40%), and is complicated by clinical ascites and/or encephalopathy within 4 weeks in patients with previously diagnosed or undiagnosed chronic liver disease or cirrhosis. The 28-day mortality associated with acute-on-chronic liver failure is high.^9^ Uncommon portal systemic collateral circulation was defined as splenorenal, gastric–renal, retroperitoneal, and cardiac angle venous shunts.^10^

### 2.4 Statistical analysis

Continuous variables are expressed as means with standard deviation and were compared using the independent sample *t* test. Categorical variables are expressed as frequencies with percentage and were compared using the chi-square test or Fisher's exact test. All tests were two sided. *p* < 0.05 was considered significant. Univariate logistic regression was performed to analyze predictors of duodenal variceal bleeding; variables with *p* < 0.30 underwent further multivariate analysis using stepwise selection in both directions. Statistical analyses were performed using R version 3.5.3 (www.r-project.org).

## 3 Results

### 3.1 Characteristics of patients and DVs

In our hospital, most patients who are admitted for endoscopy have liver disease. During the study period, 80,850 patients underwent gastroscopy for a total of 122,040 endoscopy sessions. Among these, 31,474 were diagnosed with varices. DVs were diagnosed in 52 patients (101 sessions), and eight of these exhibited duodenal variceal bleeding (15.4%).

**Fig. 1:**
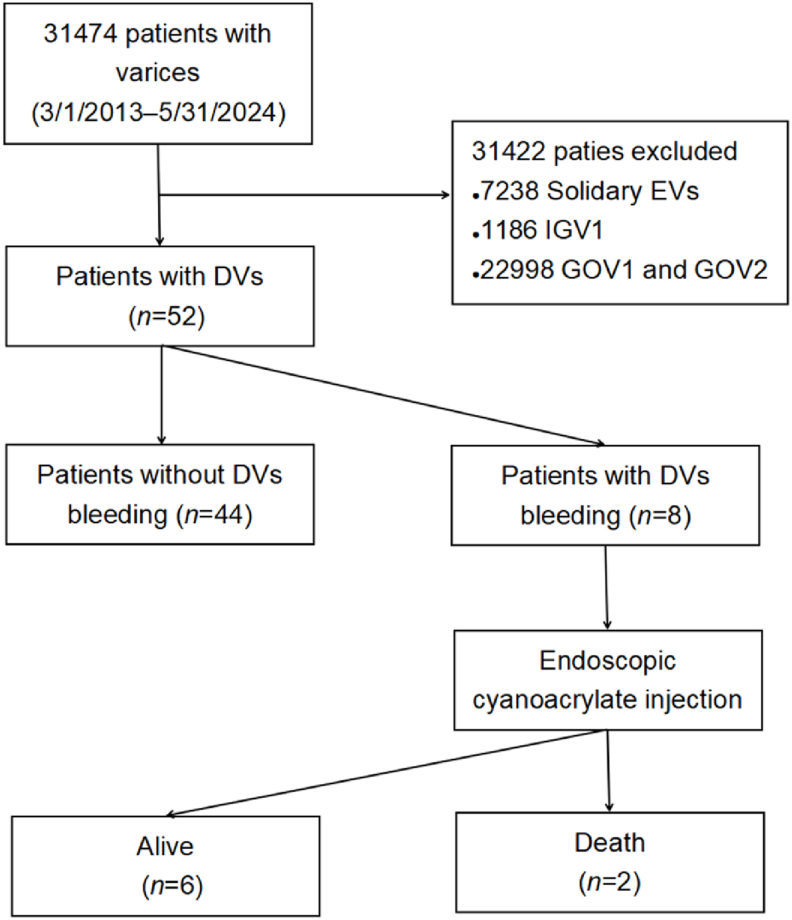
**Study flowchart.** DVs, duodenal varices; EVs, esophageal varices; GOVs, gastroesophageal varices; IGV, isolated gastric varices.

Among the 52 patients with DVs, the diagnosis was made via routine examination in 38 patients and via emergency endoscopy in 14 (Fig. 1). The characteristics of the patients and DVs are shown in Table 1. Forty-one patients were men (78.8%). The underlying cause of DVs was cirrhosis in 48 patients (92.3%). DV location was the descending segment of the duodenum in 34 (65.4%). Forty-four patients (84.6%) had concomitant esophageal and gastric varices, and 17 (32.7%) had a history of treatment for esophageal and gastric varices. The proportion of patients with hepatocellular carcinoma, liver failure, and uncommon portal systemic collateral circulation was 26.9%, 11.5%, and 25.0%, respectively. No duodenal ampullary carcinoma or cholangiocarcinoma was identified in any patient.

**Table 1:** Characteristics of the patients and duodenal varices.

Characteristics	Total (*n* = 52)	DVs without bleeding (*n* = 44)	DVs bleeding (*n* = 8)	*p*
Sex (M/F), *n* (%)	41/11 (78.8/21.2)	35/9 (79.5/20.5)	6/2 (75.0/25.0)	>0.999
Age, year, mean (SD)	52.1 (10.9)	51.7 (9.8)	54.5 (16.2)	0.506^a^
Aetiology, *n* (%)				0.425
Liver cirrhosis	48 (92.3)	40 (90.9)	8 (100.0)	
HBV	26 (50.0)	24 (54.5)	2 (25.0)	
HCV	2 (3.8)	1 (2.3)	1 (12.5)	
Alcoholic	9 (17.3)	7 (15.9)	2 (25.0)	
Alcoholic + Budd-Chiari Syndrom	1 (1.9)	1 (2.3)	0 (0.0)	
AIH	4 (7.7)	4 (9.1)	0 (0.0)	
Drug induced	1 (1.9)	1 (2.3)	0 (0.0)	
Unknown	5 (9.6)	2 (5.9)	3 (37.5)	
Non-cirrhosis	4 (7.7)	4 (9.1)	0 (0.0)	
Budd-Chiari Syndrom	2 (3.8)	2 (4.5)	0 (0.0)	
Caroli's disease	1 (1.9)	1 (2.3)	0 (0.0)	
Idiopathic	1 (1.9)	1 (2.3)	0 (0.0)	
Location of DVs, *n* (%)	52 (100.0)	44 (100.0)	8 (100.0)	0.494
Bulb	13 (25.0)	12 (27.3)	1 (12.5)	
Decending part	34 (65.4)	27 (61.4)	7 (87.5)	
Bulb and decending part	5 (9.6)	5 (11.4)	0 (0.0)	
Coexistence with GVs and EVs, *n* (%)	44 (84.6)	40 (90.0)	4 (50.0)	0.016
History of endoscopic treatments, *n* (%)	17 (32.7)	16 (36.4)	1 (12.5)	0.361
Hepatocellular carcinoma, *n* (%)	14 (26.9)	12 (27.3)	2 (25.0)	>0.999
Acute-on-chronic liver failure, *n* (%)	6 (11.5)	4 (9.1)	2 (25.0)	0.488
Uncommon portal systemic collateral circulations, *n* (%)	13 (25.0)	10 (22.7)	3 (37.5)	0.657

M/F: Male/Female; SD, standard deviation; HBV, hepatitis B virus; HCV, hepatitis C virus; AIH, autoimmune hepatitis; DVs, duodenal varices; GVs, gastric varices; EVs, esophageal varices.

a *t* = −0.670.

The DVs detection rate was 8.3 per 10,000 endoscopies. Among the patients with upper gastrointestinal varices, DVs were detected in 52 (0.165%).

Among the emergency endoscopies, 8681 sessions were associated with a diagnosis of variceal bleeding. Among the variceal bleeding sessions, nine (eight patients) were associated with duodenal variceal bleeding (0.1%).

### 3.2 Emergency treatment and follow-up of duodenal variceal bleeding

**Fig. 2:**
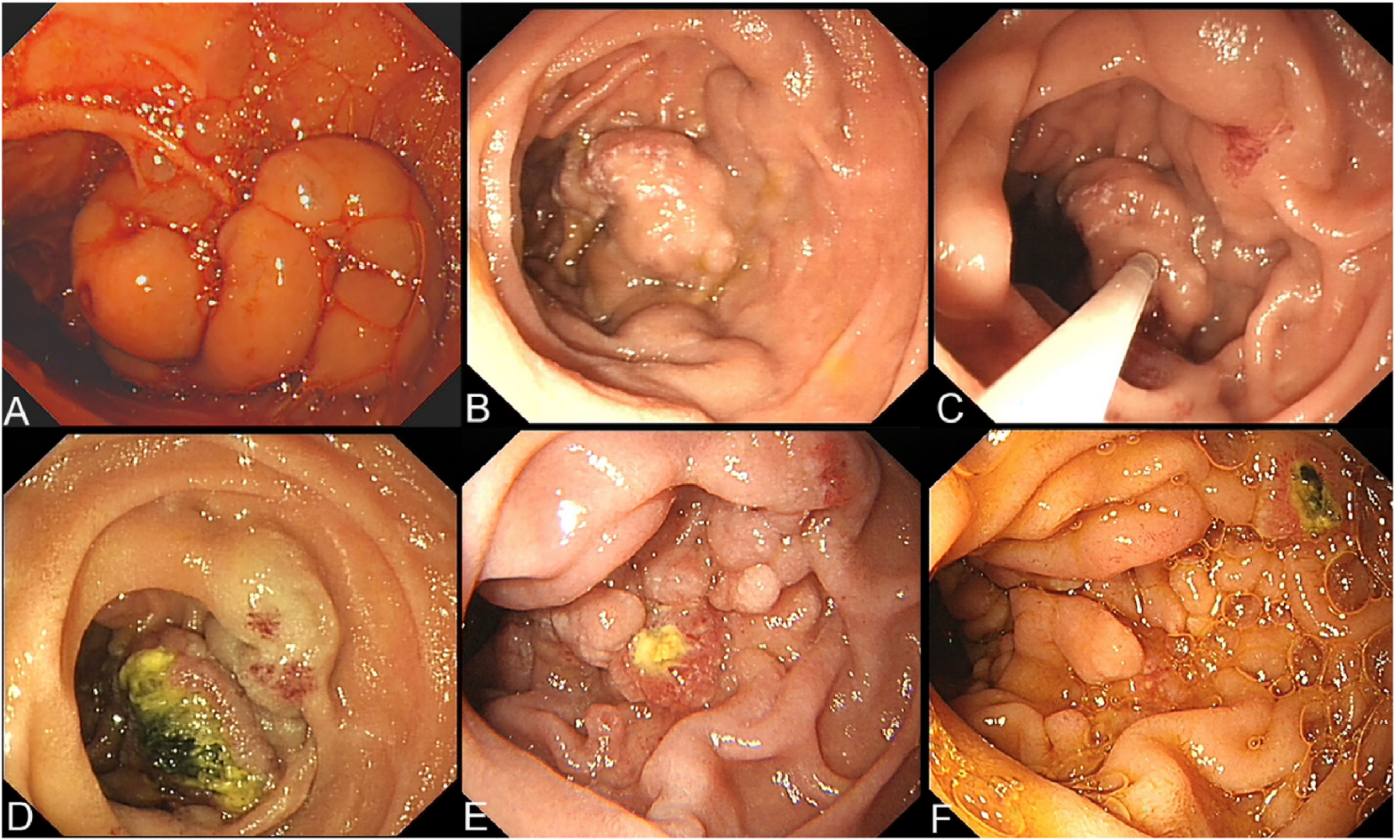
**Endoscopic cyanoacrylate injection for duodenal variceal bleeding**. **A**, Emergency endoscopy showed variceal erosion in the descending duodenum. **B**, Endoscopic cyanoacrylate injection for secondary prophylaxis. **C**, One week after secondary prophylaxis, ulceration and redness were visualized. **D**, Six weeks after secondary prophylaxis, glue expulsion and food residue are apparent. **E**, Fifteen weeks after secondary prophylaxis, there is granular tissue and expulsion of the glue. **F**, The varices disappeared 7 months later. DVs, duodenal varices.

Eight patients with acute duodenal variceal bleeding underwent emergency endoscopic treatment; cyanoacrylate injection was performed in all (Fig. 2). The characteristics of these eight patients (numbered in chronological order of admission) are shown in Table 2. The bleeding location was descending duodenum in all. The rates of 6-week mortality, successful hemostasis, and rebleeding were 12.5%, 100.0%, and 12.5%, respectively. No ectopic embolism was observed after injection in any patient.

**Table 2:** Summary of eight patients who underwent emergency endoscopic cyanoacrylate injection for duodenal variceal bleeding.

Case	Sex	Age (y)	Cause	Child–Pugh class	Comorbidities	Endoscopic findings	Dose of CA (mL)	Rebleeding time (d)	Adverse events	Additional injection of CA (mL)	Outcome
1	F	66	HCV	C	LF	Erosion	3.0	–	–	–	Death (bleeding)
2	M	54	Alcoholic	B	AKI	Red plug	1.5	–	–	2.0^a^	Alive
3	M	69	Alcoholic	C	–	Spurting blood	1.5	–	–	3.0^a^	Alive
4	M	59	HBV	A	HCC	Erosion	2.5	–	Fever	–	Alive
5	F	68	Unknown	C	HCC, ACLF, SH	Red plug	1.5	11	–	1.5^b^	Death (bleeding)
6	M	49	HBV	B	HCC	Red plug	1.0	–	Fever	–	Alive
7	M	19	Unknown	A	–	Red plug	1.0	–	–	–	Alive
8	M	52	Unknown	B	–	Erosion	1.0	–	–	–	Alive

ACLF, acute-on-chronic liver failure; AKI, acute kidney injury; HCC, hepatocellular carcinoma; SH, subdural hematoma; CA, cyanoacrylate.

a Secondary prophylaxis.

b Emergency endoscopic treatment.

Patient 5 presented with melena 11 days after emergency endoscopic treatment. After repeat endoscopic cyanoacrylate injection, the melena had not been relieved. Two days later, she died of upper gastrointestinal bleeding. Her comorbidities included liver failure, liver cancer, hepatic encephalopathy, and traumatic subdural hematoma.

Fever occurred in patients 4 and 6 after emergency endoscopic treatment. The fever resolved after 5 and 7 days of intravenous ceftriaxone administration, respectively. Patient 6 had undergone hepatic artery embolization for hepatocellular carcinoma (Barcelona Clinic Liver Cancer stage C/China Liver Cancer stage IIIb) the day before duodenal variceal bleeding occurred.

Patient 1 presented with melena 88 days after emergency endoscopic treatment. She also had symptoms of hepatic encephalopathy owing to liver failure and had been diagnosed with acute kidney injury. Although emergency esophagogastroduodenoscopy was performed twice and colonoscopy once, no bleeding site was found. The cause of melena was presumed to be small bowel bleeding; however, no further examination was performed because her vital signs were unstable. The patient died of gastrointestinal bleeding 8 days after admission.

### 3.3 Secondary prophylaxis for duodenal variceal bleeding

Only two patients with Child‒Pugh class A cirrhosis received secondary prophylaxis. Both had DVs located in the descending segment of the duodenum and one had undergone an emergency endoscopic cyanoacrylate injection for duodenal variceal bleeding 5 months prior. Neither patient experienced an adverse event (Fig. 2).

### 3.4 Predictors of duodenal variceal bleeding

Univariate logistic regression showed that age >65 years, other coexisting varices, serum albumin concentration, blood urea concentration, and Child–Pugh score were significant predictors of duodenal variceal bleeding. In the multivariate analysis, age >65 years and low hemoglobin concentration were independent predictors of duodenal variceal bleeding (Table 3).

**Table 3:** Logistic regression analysis of potential predictors of duodenal variceal bleeding.

Variable	Univariate			Multivariate		
	OR	95% CI	*p*	OR	95% CI	*p*
Sex (male vs. female)	0.77	0.13–4.48	0.770			
Age (y) (>65 vs. ≤65)	8.2	1.28–52.16	0.026	24.66	1.35–448.92	0.030
Etiology (virus vs. non-virus)	0.38	0.08–1.79	0.220			
Endoscopic treatment history (absent vs. present)	0.25	0.03–2.22	0.210			
Hepatocellular carcinoma (absent vs. present)	0.88	0.16–5.02	0.890			
ACLF (absent vs. present)	3.33	0.49–22.33	0.215			
Coexistence with other varices (absent vs. present)	10	1.78–56.15	0.008			
Uncommon portal systemic collateral circulations (absent vs. present)	2.04	0.41–10.06	0.381			
Systolic blood pressure (mm Hg) (≥90 vs. <90)	0.00	0–inf	0.990			
Pulse (per min) (≥100 vs. <100)	4.55	0.63–33.11	0.130			
Albumin (g/L)	0.77	0.64–0.93	0.006			
Total bilirubin (umol/L)	1.01	0.99–1.03	0.200			
INR (>1.5 vs. ≤1.5)	2.11	0.34–12.99	0.420			
Hemoglobin (g/L)	0.93	0.87–0.99	0.210	0.91	0.85–0.98	0.016
Blood urea (mmol/L) (>10 vs. ≤10)	5.28	1.06–26.28	0.040			
CTP	1.49	1.05–2.11	0.023			

OR, odds ratio; CI, confidence interval; ACLF, acute-on-chronic liver failure; INR, international normalized ratio; CTP, Child-Turcotte-Pugh score; inf, infinite.

## 4 Discussion

In this single-center retrospective study, the prevalence of DVs was 0.08%.DVs accounted for 0.16% of upper gastrointestinal varices, and duodenal variceal bleeding was present in 15.4% of patients with DVs. Furthermore, DVs were mainly found in men (78.8%) and a large majority were associated with liver cirrhosis (92.3%). Co-occurrence with esophageal and gastric varices was quite common (84.6%), and most were located in the descending segment of the duodenum (65.4%). Successful hemostasis was achieved at the time of endoscopy in all cases of emergency endoscopic duodenal varicose cyanoacrylate embolization. Six-week mortality was 12.5% and the rebleeding rate was 12.5%. Logistic regression analysis revealed that age >65 years and low hemoglobin concentration were independent predictors of duodenal variceal bleeding.

DVs are rare and data regarding their incidence varies between studies because of differences in populations and hospital admission policies. Because most patients with DVs in our hospital have liver disease and formation of DVs may be related to liver disease, we believe that the prevalence of DVs in our hospital may be greater than that reported in previous studies. However, our detection rate of DVs is similar to that reported in previous studies^2^^,^^11^; this may be because DVs have thicker vein walls or are indistinguishable from mucosal folds because of inconspicuous bulges. In emergency endoscopy, the presence of blood in the duodenum may hinder their detection. Therefore, blood should be flushed away for a complete duodenal examination. If the diagnosis of DVs is difficult, endoscopic ultrasonography may be helpful.^12^

DVs are usually associated with portal hypertension, especially liver cirrhosis. In our study, most DVs were located in the descending segment, which is consistent with previous reports.^2^^,^^13^

Esophagogastric variceal bleeding is one of the main complications of portal hypertension in patients with cirrhosis, with reported mortality rates in hospitalized patients ranging from 30% to 50%.^14^ Although bleeding from DVs is infrequent, it is typically massive and often fatal; the mortality approaches 40%.^7^ Endoscopic band ligation, tissue adhesives, and transjugular intrahepatic portal systemic shunting are recommended for treating esophagogastric variceal bleeding.^3^^,^^14^ However, there are no guidelines for treating duodenal variceal bleeding because of its low incidence and the lack of data from randomized controlled trials. A recently published systemic review reported that endoscopic treatment of duodenal variceal bleeding (including sclerotherapy, band ligation, tissue adhesive, and combination therapy) can achieve high rates of hemostasis at the time of endoscopy and treatment success and low rates of rebleeding and mortality.^7^ The authors suggested that cyanoacrylate injection may be better for DVs than other endoscopic treatment options.^7^ In our study, the rate of successful hemostasis at the time of endoscopic cyanoacrylate injection was higher than that reported for endoscopic tissue adhesive in the meta-analysis (100.0% vs. 94.7%).

DVs frequently communicate with the systemic circulation through the pancreaticoduodenal veins,^15^ and cyanoacrylate embolization may cause ectopic embolism, leading to cerebral infarction or atrial embolism.^16^^,^^17^ When the cyanoacrylate injection dose is high, partial blockage of the duodenal cavity may be caused by the expulsion of glue.^5^ On the basis of these concerns, we believe that when the circulation surrounding the DVs is unknown, the injected dose should be aimed at achieving hemostasis rather than complete embolization. Secondary prophylaxis could be considered if further imaging reveals no obvious communication between the systemic circulation and the DVs. If significant systemic circulation communication is present, balloon-occluded retrograde transvenous obliteration may be considered,^5^^,^^15^ as it helps reduce the risk of ectopic embolism.

Two of our patients experienced rebleeding after emergency treatment. Possible reasons for rebleeding in these two include age >65 years, poor liver function (Child‒Pugh class C, liver failure), and liver cancer, which are important components in well-established scoring systems^18^ for predicting rebleeding events, such as the Rockall score,^19^ AIMS65 score^20^ and Blatchford score.^21^ For patients who are at high risk of rebleeding and who are unfit for secondary prophylaxis in the short term, preventing rebleeding is a difficult issue. The benefits and risks of adequate embolization should be considered with caution in emergency endoscopic treatment.

The two patients who received secondary prophylaxis achieved satisfactory treatment effects, and there were no adverse events or rebleeding during follow-up. This may be attributed to their stable condition before treatment. These findings suggest that secondary prophylaxis with endoscopic cyanoacrylate embolization can achieve a better curative effect in stable patients with DVs. Considering the risk of rebleeding, secondary prophylaxis should be performed as early as possible. Although our analysis showed that age >65 years and low hemoglobin concentration were predictors of duodenal variceal bleeding, the sample size was small and our results should be interpreted cautiously.

This study has several limitations: it is retrospective, and only a subset of patients received endoscopic treatment, which may affect treatment efficacy and generalizability. Incomplete information about outpatient patients may also compromise the accuracy of the analyses. Additionally, as the study was conducted at a single center, the results may lack broad representativeness and could be influenced by the specific procedures and diagnostic criteria used.

## 5 Conclusions

DVs are uncommon, even in hospitals where liver disease is prevalent. Emergency endoscopic cyanoacrylate embolization appears to be highly effective in treating duodenal variceal bleeding. When performing emergency endoscopy, complete vein embolization may be considered for patients in poor condition. Endoscopic secondary prophylaxis has achieved good results. Age >65 years and low hemoglobin concentration are predictors of duodenal variceal bleeding.

## CRediT authorship contribution statement

**Jin-dong Chu:** Writing – original draft, Formal analysis, Data curation, Methodology, Visualization. **Qian Bi:** Writing – review & editing, Funding acquisition, Formal analysis. **Yan-ling Wang:** Investigation, Visualization. **Xue-mei Ma:** Investigation. **Bo Liu:** Investigation. **Liang Wu:** Investigation. **Shuai Wang:** Investigation. **Li-jun Shen:** Investigation. **Xiao-bao Qi:** Supervision. **Zheng Lu:** Writing – review & editing, Funding acquisition, Conceptualization, Methodology, Project administration.

## Informed consent

All patients provided written informed consent.

## Data availability statement

The data from this study are available from the corresponding author on reasonable request.

## Ethics statement

The study was approved by the institutional review board of the Fifth Medical Center of Chinese PLA General Hospital (KY-2024-7-98-1).

## Funding

This study was supported by grants from the 10.13039/100014717National Natural Science Foundation of China (No. 82270694) and the Beijing Nova Program (#20220484197).

## Declaration of Generative AI and AI-assisted technologies in the writing process

Not applicable.

## Declaration of competing interest

The authors and funders have no conflicts of interest to declare.
